# P-266. Does routine use of sporicidal disinfectants for all post-discharge hospital rooms reduce environmental contamination with *Clostridioides difficile* spores?

**DOI:** 10.1093/ofid/ofae631.470

**Published:** 2025-01-29

**Authors:** Sarah Plumlee, Jennifer Cadnum, Martin Varghese, Rebecca Bartles, Samir Memic, Annette Jencson, Curtis Donskey

**Affiliations:** Association for Professionals in Infection Control and Epidemiology, Prairieville, Louisiana; Northeast Ohio VA Medical Center, Cleveland, Ohio; Louis Stokes VA Medical Center, Cleveland, Ohio; APIC, Maple Valley, Washington; Northeast Ohio VA Healthcare System, Cleveland, Ohio; Cleveland VA Hospital, Cleveland, Ohio; Cleveland VA Hospital, Cleveland, Ohio

## Abstract

**Background:**

Asymptomatic carriage of *Clostridioides difficile* is common in healthcare facilities. Routine use of sporicidal disinfectants for all post-discharge rooms has been proposed as a measure to address environmental shedding by carriers, but limited information is available on the effectiveness of this approach.

Recovery of C. difficile from CDI and non-CDI rooms in hospitals using sporicidal disinfectants only in CDI rooms versus in all post-discharge rooms

**Figure:**
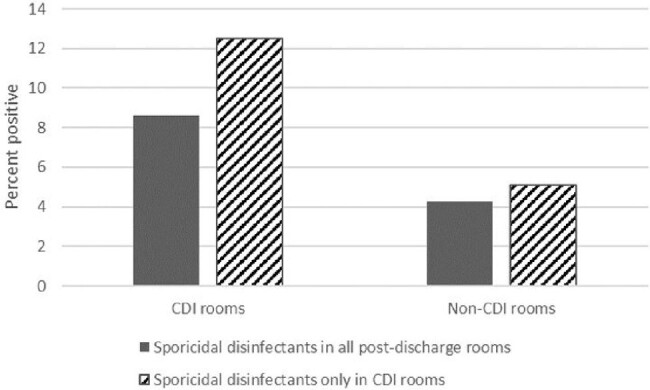

**Methods:**

We conducted a culture survey to assess environmental contamination with *C. difficile* in patient rooms after cleaning and disinfection in 30 acute care hospitals, including 17 (57%) using sporicidal disinfectants (11 peracetic acid-based products and 6 chlorine-based products) for all post-discharge rooms and 13 (43%) using sporicidal disinfectants (1 peracetic acid-based product and 12 chlorine-based products) only for *C. difficile* infection (CDI) rooms. Cultures for *C. difficile* were collected from 10 rooms per hospital (up to 3 CDI rooms), including high-touch surfaces in the room (N=10 sites) and in the bathroom (N=10 sites). A mixed-effects logistic regression model was used to compare contamination in non-CDI (primary outcome) and CDI rooms in facilities using sporicidal disinfectants in non-CDI and CDI rooms versus only in CDI rooms.

**Results:**

Of 595 total patient rooms and bathrooms cultured, 339 (57%) were in hospitals using sporicidal disinfectants for all post-discharge rooms and 256 (43%) were in hospitals using sporicidal disinfectants only in CDI rooms. As shown in Figure 1, there was a trend toward reduced *C. difficile* contamination in non-CDI and CDI rooms in facilities using sporicidal disinfectants in all post-discharge rooms versus only in CDI rooms, but the difference was not statistically significant (P>0.62).

**Conclusion:**

In a survey of 30 hospitals, use of sporicidal disinfectants for all post-discharge rooms was not associated with a statistically significant reduction in *C. difficile* contamination in non-CDI rooms. Additional studies are needed to determine if interventions to improve the effectiveness of sporicidal disinfectant application will reduce *C. difficile* contamination in real-world settings.

**Disclosures:**

**Curtis Donskey, MD**, Clorox: Grant/Research Support|Pfizer: Grant/Research Support

